# Carbon dioxide conversion to fuel over alumina-supported ruthenium catalysts

**DOI:** 10.1016/j.heliyon.2024.e31349

**Published:** 2024-05-18

**Authors:** Galeboe Ralengole, Kalala Jalama, Phathutshedzo Khangale

**Affiliations:** a Department of Chemical Engineering, Faculty of Engineering and the Built Environment, University of Johannesburg, Doornfontein, 2028, Johannesburg, South Africa; b Institute for Catalysis and Energy Solutions, College of Science, Engineering and Technology, University of South Africa, Johannesburg, South Africa

**Keywords:** Ru/Al_2_O_3_, Ru-based catalysts, CO_2_ hydrogenation, Potassium, Pressure, Temperature

## Abstract

In this study, ruthenium-based catalysts were prepared for CO_2_ hydrogenation. Incipient-wetness-impregnation of the alumina-support with ruthenium (III) nitrosyl nitrate solution to achieve 0.5 wt% Ru loading on supports was used to prepare these catalysts. Potassium (0–3 % wt%) was used to further promote the catalysts. TPR, CO_2_-TPD, XRD, TEM, XPS, SEM, and EDS analyses were used to characterize catalyst properties. The hydrogenation of CO_2_ catalytic tests were conducted and the effect of operating conditions (temperature, pressure, and space velocity) were investigated. These studies were conducted in a tubular fixed-bed reactor. The CO_2_ conversion over these catalysts was found to be low and the dominating product observed was CH_4_ with a small amount of C_2+_ forming when K was added to the catalyst. The optimum potassium loading for improved C_2+_ product yield over Ru/Al_2_O_3_ was 1%K for CO_2_ hydrogenation.

## 1 Introduction

Fossil fuels and chemical feedstocks with a wide carbon number distribution are in greater demand. The Fischer-Tropsch Synthesis (FTS) appears to be capable of satisfying this need. However, it emits large quantities of carbon dioxide gas leading to the global warming phenomenon [1]. An increasing number of studies are investigating carbon dioxide hydrogenation to fuels [2]. Production of renewable hydrogen through electrolysis is being touted as a key enabler; when CO_2_ sorption technologies are combined with solar-based H_2_ production, the CO_2_ conversion might be accomplished sustainably [1]. This will contribute to carbon capture and utilization (CCU), which has significantly surpassed carbon capture and storage (CCS) in recent years. As a result, it is a great opportunity to study and investigate the CO_2_ conversion to fuels and other useful chemicals [3]. With this being said, great research work is being done with some noble metal-based catalysts, including Ruthenium, Palladium, and Platinum, already being studied for their catalytic properties in modified FTS. These catalysts were reported to have potential to promote hydrogenation of CO_2_ at the lowest temperature range (170–270 °C); however, due to their high costs, they have not received much attention for CO_2_ hydrogenation in recent years despite having been reported to have shown good potential for selectivity of other hydrocarbons such as C_2_H_4_, C_3_H_3_, CH_2_O, and CH_3_OH, including CO which could be manipulated to produce FTS products [4]. This work evaluates the effect of operating conditions (temperature, pressure, and space velocity) on CO_2_ conversion to hydrocarbons over alumina-supported ruthenium-based catalysts.

## 2 Experimental details

### 2.1 Catalyst preparation and characterization

The gamma alumina-support (ɣ-Al_2_O_3_) which was supplied by Sigma Aldrich was prepared by mixing with distilled water followed by drying in air at 100 °C for 12 h and then calcined in air at 400 °C for 4 h. Alumina-supported ruthenium catalysts were prepared from Ruthenium (III) nitrosyl nitrate (Ru(NO) (NO_3_)_3_ solution supplied by Sigma Aldrich. Following calcination of the alumina support, the ruthenium-based catalysts were synthesized by incipient wetness impregnation of the alumina support with (Ru(NO) (NO_3_)_3_ aqueous solution to give 0.5 wt% Ru in Al_2_O_3_. The impregnated support was dried in air at 100 °C for approximately 12 h followed by calcination in air at 400 °C for 4 h. Potassium promoter was added to the catalyst by subsequent incipient wetness impregnation method. A solution of anhydrate potassium nitrate (KNO_3_) was prepared by mixing with distilled water and then the Ru catalyst was impregnated with this solution. The impregnated support was dried in air at 100 °C, for 24 h and then calcined in air at 600 °C for 5 h. The targeted content of K in the Ru/Al_2_O_3_ catalyst was 1 %, 2 %, and 3 % respectively.

Temperature Programmed Reduction (TPR) analysis was performed using a unit constructed in our laboratory to study the reduction behavior of the catalysts, where about 100 mg of calcined catalyst samples were loaded in a reactor and degassed using argon as an inert gas flowing at 30 ml/min. Temperature was held at 300 °C for 60 min and then allowed to cool down to room temperature. Before TPR was started, the Ar flow was replaced with 5%H_2_/Ar. The temperature was elevated to 700 °C at a heating rate of 10 ^°^C/min. TCD was also located at the reactor outlet to measure reducing gas concentration. The reactor was also fitted with a thermocouple that measured reaction temperature and the data was recorded using a data logger (PecoLog Recorder ADC-20, serial number AQ253/036).

The ruthenium phase and the structure of the catalyst before and after reduction were determined using X-ray Diffraction (XRD) analysis. The Rigaku Ultima IV X-ray powder diffractometer with PDXL analysis software was used to conduct the analysis. The analysis parameters used were Cu-Kα (λ = 1.54 Å) radiation source, current and voltage set at 40 mA and 40 kV and step width of 2θ = 0.017°.

CO_2_-Temperature Programed Desorption (CO_2_-TPD) was conducted to determine the basicity of the catalysts. The same apparatus which was used to perform TPR was also used to perform CO_2_-TPD. This was done in two steps. Firstly, the catalysts were reduced at 230 °C for 16 h under a flow of H_2_ gas using the same reactor used for FT synthesis, then allowed to cool down to room temperature at which passivation was done for 2 h using 5%O_2_/He. The second step was to conduct CO_2_-TPD. This was done by transferring 200-mg of the reduced and passivated catalysts into a tubular reactor used as described in TPR. The catalysts were firstly degassed with a flow of pure He gas at 300 °C for 60 min at a flow rate of 30 ml/min at a heating rate of 10 ^°^C/min and then allowed by cooling to room temperature. Following this, the catalysts were reduced with a flow of 5%H_2_/Ar at 230 °C for 30 min at a heating rate of 10 ^°^C/min. The flow was then switched to He at 230 °C (30 ml/min) for a further 30 min before cooling the reactor to 50 °C. The temperature was maintained at 50 °C for 10 min and then the gas was switched to 10%CO_2_/He for 60 min. Following this step, the flow was switched to pure He and this step was necessary to remove all molecules that were physically adsorbed on the sample. The TCD signal was stabilized and then the CO_2_-TPD was performed using He gas by increasing the temperature from 50 °C to 700 °C at a heating rate of 5 ^°^C/min and then the temperature was maintained at 700 °C for 30 min. The results of CO_2_-TPD were recorded using PecoLog as described on TPR section above.

The X-ray photoelectron spectroscopy (XPS) analysis was carried out at the Physics Labs of the University of Johannesburg, Auckland Park Kingsway campus. The ultra-high vacuum (UHV) chamber used for the XPS spectra presented in this work had a base pressure of 2 × 10-10 mbar at ambient temperature. A SPECS PHOIBOS 150 hemispherical electron energy analyzer and a SPECS XR 50 M monochromatized X-ray source with an Al anode (Al K excitation line, or hv = 1486.71 Ev) were installed in the UHV chamber. The combined analyser and photon source system's spectra's overall energy resolution was set to 0.9 for survey scans and 0.6 Ev for core level spectra. By illuminating the samples with a low-energy electron beam produced by a flood gun set to run at the following parameters: electron energy = 2.5 Ev, electron flux = 20 μA, the surface charging of the samples was compensated. The XPS data was cleaned up and replotted in an excel file. By changing the oxidic O1s binding energy to 531.5 eV, the findings were rectified.

Transmission electron microscopy (TEM) analysis were carried out to study the shape and particle size of the catalysts. All Ru/Al_2_O_3_ samples were subjected to TEM using a Joel JEM-2100F transmission electron microscopy system with a LaB6 source at an acceleration voltage of 200 kV. Small amounts of synthetic ruthenium-based catalysts were dropped onto a Cu-grid TEM grid (200 mesh size) that had been covered in a lacy carbon layer to create the TEM samples. Using a digital charge coupled device (CCD) camera attached to the transmission electron microscope, pictures of mesoporous ruthenium oxides were taken.

The scanning electron microscyopy (SEM) analsis were carried out to study the surface roughness, pores, and Ru and support particle shape of the catalysts. This unit uses a Tuscan Vega 3 LMH which is operated at 20 kV accelerating voltage, using secondary electron detector (SE) and also equipped with energy dispersive spectroscopy (EDS). Materials were initially carbon-coated with the Agar Turbo Carbon coater to improve their conductivity.

### 2.2 Catalyst testing

CO_2_ hydrogenation was studied in a continuous reactor tube with internal diameter of 16 mm and length of 220 mm. The reactor was loaded with 500 mg of catalyst particles using glass wool to keep the catalyst at the center of the reactor. The reactor was fitted with a thermocouple (thermon type “K” with the length of 400 mm and 1.5 mm diameter) and the temperature was controlled using a Unitemp temperature control unit. The additional thermocouple that measured the core temperature was also fitted. A back-pressure regulator was also fitted on the outlet of the reactor to regulate the desired operating pressure. A condensed products trap was also fitted downstream of the reactor. A three-way valve was installed downstream of the back-pressure regulator to direct outlet flow either to vent or to the online Gas Chromatograph (GC) for analysis of product gasses. On the feed, the reactor was fitted with Aalborg GFC17 mass flow controller (MFC) to regulate the volumetric flow of the feed gas. A heating element was fitted to the reactor and insulation was installed to preserve the heat loss.

The product gas flow was sent to an online gas chromatograph [DANI Master GC], where the product gases was analyzed. The analysis was done by means of a flame ionization detector (FID) connected to a fused silica capillary column (30 m long with 0.32 mm diameter) and a thermal conductivity detector (TCD) connected to a carboxen 1000 column. The carrier gasses used were Hydrogen (H_2_) for the FID and Argon (Ar) for the TCD. Clarity Apex software was used to process GC data.

In a typical run, 500 mg of catalyst was weighed and loaded into a fixed-bed reactor. The catalyst was activated by increasing the temperature from room temperature to 230 °C and maintained at this temperature for 15 h using hydrogen as a reduction gas at atmospheric pressure. The reactor was allowed to cool to room temperature before the feed gas (66.9 % H_2_, 23.2 % CO_2_, and 9.9 % N_2_) was introduced. The operating conditions were varied to study the effect of temperature, pressure, and space velocity.

The hydrocarbon distribution was calculated based on the total carbon moles with the unit of C-mole% on all evaluated catalysts and the equations used are listed in prior studies from our lab [5].

N_2_ was used in the reaction feed gas to serve as an internal standard that allowed us to take gas expansion into account for accurate calculations of the CO_2_ conversion.

## 3 Results and discussion

### 3.1 Catalyst characterization

#### 3.1.1 Hydrogen Temperature Programmed Reduction (H-TPR) analysis

**Fig. 1:**
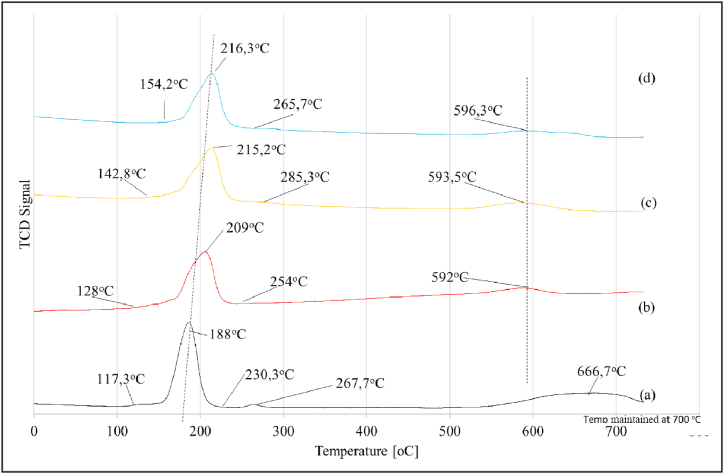
H_2_-TPR data for (a) 0.5%Ru/γ−Al_2_O_3_; (b) 0.5%Ru/γ−Al_2_O_3_–1%K; (c) 0.5%Ru/γ−Al_2_O_3_–2%K, and (d) 0.5%Ru/γ−Al_2_O_3_–3%K.

The results of H_2_-TPR study on alumina-supported Ru-based catalysts are shown in Fig. 1. The unpromoted Ru-catalyst results were represented in Fig. 1a, while the results of the promoted catalysts were represented as Fig. 1b showing Ru-catalyst promoted with 1 % of potassium; Fig. 1c showing Ru-catalyst promoted with 2 % of potassium, and Fig. 1d showing Ru-catalyst promoted with 3 % of potassium.

For the unpromoted alumina-supported ruthenium catalyst (Fig. 1a), the first peak, which is the largest, appears from 117 °C and shows its maximum at ca. 188 °C and then ends at 230 °C. The second but very small peak was also observed with its maximum at 268 °C before the last extended peak is observed with a maximum at about 667 °C. The first broad hydrogen reduction peak was attributed to RuO_2_ reduction to Ru metallic state, while the small peak with its maximum at 268 °C could be associated with the reduction of ruthenium species which were in strong interaction with the Al_2_O_3_ support [6,7]. The peak observed at 667 °C could represent reduction of some ruthenium species which are in strong interaction with the alumina support. It can also be observed that when the catalyst was promoted with potassium, (Fig. 1 b-d), the two-step reduction processes of ruthenium were represented by one peak. Potassium addition shifted the peaks more to higher temperatures, i.e. 209 °C, 215 °C and 216 °C for catalysts promoted with 1%K, 2%K and 3%K, respectively. Moreover, the peak observed at higher temperatures shifted to a lower temperature when potassium was added compared to the unpromoted catalyst indicating that potassium may have limited strong interaction between the support and ruthenium. This data suggests that the more potassium promoter is added, the higher the interactions between some ruthenium species and the alumina support.

#### 3.1.2 Carbon-dioxide temperature programmed desorption (CO_2_-TPD) analysis

**Fig. 2:**
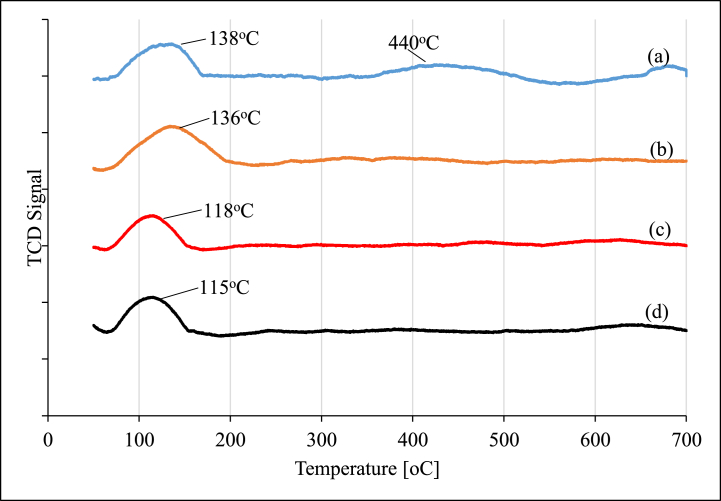
CO_2_-TPD data for (a) 0.5%Ru/γ−Al_2_O_3_; (b) 0.5%Ru/γ−Al_2_O_3_–1%K; (c) 0.5%Ru/γ−Al_2_O_3_–2%K, and (d) 0.5%Ru/γ−Al_2_O_3_–3%K.

The CO_2_-TPD profiles of pre-reduced and passivated Ruthenium catalysts supported on alumina are presented in Fig. 2.

The unpromoted catalyst displayed two desorption peaks at 138 °C and 440 °C (Fig. 2a). When the catalyst was promoted with 1 % of potassium (Fig. 2b), only one desorption peak was observed at ca. 136 °C. As the potassium content was increased to 2 wt% (Fig. 2c), once again only one desorption peak was observed at ca. 118 °C. Further addition of potassium to 3 % (Fig. 2d), also displayed one desorption peak at ca. 115 °C. The high temperature CO_2_ desorption peak disappeared when K was added to the catalyst. This suggests that the addition of K in the Ru/Al_2_O_3_ catalyst led to complete removal of residual salt species dispersed on the alumina after calcination in air at 400 °C. The addition of potassium shifted the CO_2_ desorption peaks to slightly lower temperature at 1 % K addition and significantly lower at 2 and 3 % K. The first asymmetric peak is attributed to the CO_2_ adsorption on the basic sites of γ-Al_2_O_3_ [8]. The second peaks observed around 440 °C on the unpromoted catalyst could be due to residual salts used for catalyst preparation and which were not fully decomposed during catalyst calcination and reduction processes.

#### 3.1.3 X-ray diffraction (XRD) analysis

**Fig. 3:**
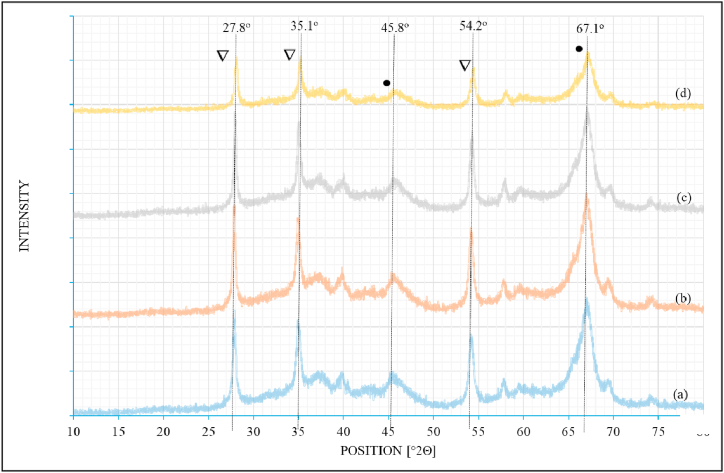
XRD data for (a) 0.5%Ru/γ−Al_2_O_3_; (b) 0.5%Ru/γ−Al_2_O_3_–1%K; (c) 0.5%Ru/γ−Al_2_O_3_–2%K, and (d) 0.5%Ru/γ−Al_2_O_3_–3%K. (•) γ-Al_2_O_3_, (∇) RuO_2_.

The XRD profile for unpromoted freshly calcined 0.5%Ru/Al_2_O_3_ catalyst is shown in Fig. 3a while the XRD profiles for the promoted catalysts were represented as Fig. 3b showing Ru-catalyst promoted with 1 % of potassium; Fig. 3c showing Ru-catalyst promoted with 2 % of potassium, and Fig. 3d showing Ru-catalyst promoted with 3 % of potassium.

All catalysts displayed diffraction peaks at 27.9°, 35.1°, 45.8°,54.3°, and 67.1° (Fig. 3 (a – d). It can also be noted that the intensity of the peaks is higher on the unpromoted catalyst and decreases with increasing promoter loading. The first, second, and fourth diffraction peaks display RuO_2_ pattern and are characteristic of tetragonal RuO_2_ [9]. The third and fifth broad peaks can be assigned to γ-Al_2_O_3_ support [8,10].

**Fig. 4:**
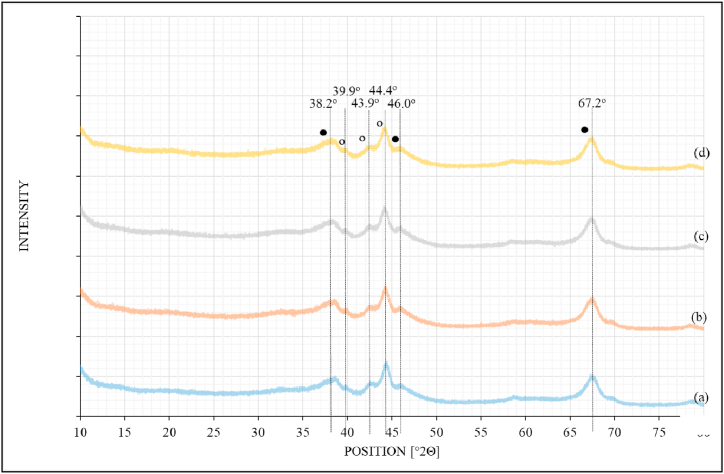
XRD data for (a) 0.5%Ru/γ−Al_2_O_3_; (b) 0.5%Ru/γ−Al_2_O_3_–1%K; (c) 0.5%Ru/γ−Al_2_O_3_–2%K, and (d) 0.5%Ru/γ−Al_2_O_3_–3%K. (•) γ−Al_2_O_3_, (ο) Ru^0^.

Fig. 4 shows the XRD profiles for the reduced catalysts supported on alumina and promoted with potassium. All catalysts were reduced with pure H_2_ flowing at 30 ml/min and at a temperature of 230 °C and passivated with 5%O_2_/He at room temperature for 2 h before removed from the reactor. Fig. 4a shows the unpromoted Ru-catalyst; Fig. 4b showing Ru-catalyst promoted with 1 % of potassium; Fig. 4c showing Ru-catalyst promoted with 2 % of potassium, and Fig. 4d showing Ru-catalyst promoted with 3 % of potassium.

Following catalyst reduction at 230 °C, diffraction angles for metallic Ru were detected at 39.9, 43.9 and 44.4° (Fig. 4 (a – d)).

The Scherrer equation (equation (1)) was applied to the XRD data to calculate the particle sizes of RuO_2_ and Ru° in all catalysts. The results obtained are summarized in Table 1 below.

**Table 1:** Catalyst particle size estimated by XRD.

	Particle Size [nm]	
Catalysts	Fresh Catalyst RuO2	Reduced Catalyst Ru
**0.5%Ru/Al**_**2**_**O**_**3**_	2.13	1.35
**0.5%Ru/Al**_**2**_**O**_**3**_**/1%K**	2.13	1.35
**0.5%Ru/Al**_**2**_**O**_**3**_**/2%K**	2.13	1.35
**0.5%Ru/Al**_**2**_**O**_**3**_**/3%K**	2.12	1.35

The Scherrer equation is expresses as:

(1) $$d=\frac{k\lambda }{\beta \;\cos\;\theta }$$

where:

d = crystallites size (nm).

k = 0.9 (Scherrer constant).

λ = 1.154056 nm (wavelength of the X-ray sources).

β = full width at half maximum (FWHM) (radians).

θ = Peak position (radians).

The average particle size of RuO_2_ on the fresh calcined alumina-supported catalyst was calculated to be 2.13 nm (Table 1) and did not change with the addition of K promoter. When the catalyst was reduced with pure H_2_ at 230 °C, only metallic Ru was observed with the decreased average particle size of 1.35 nm. The reduction in the particle size was due to the contraction of the unit cell volume of metallic Ru with respect to its oxide, Cimino et al. (2020:198).

#### 3.1.4 Transmission electron microscopy (TEM) analysis

**Fig. 5:**
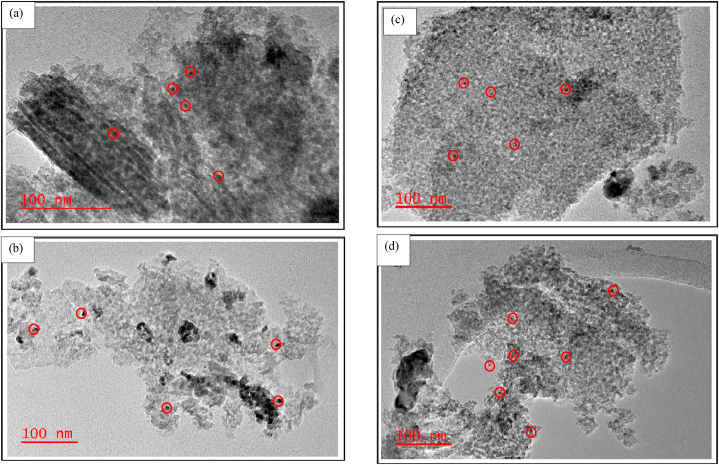

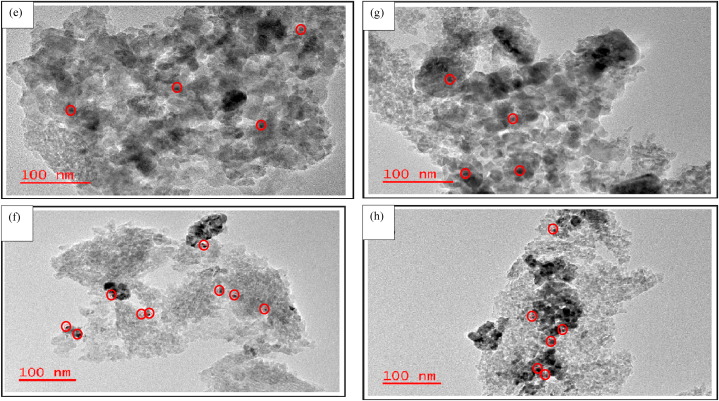
TEM data for (a) fresh 0.5%Ru/Al_2_O_3_, (b) reduced 0.5%Ru/Al_2_O_3_, (c) fresh 0.5%Ru/Al_2_O_3_/1%K, (d) reduced 0.5%Ru/Al_2_O_3_/1%K, (e) fresh 0.5%Ru/Al_2_O_3_/2%K, (f) reduced 0.5%Ru/Al_2_O_3_/2%K, (g) fresh 0.5%Ru/Al_2_O_3_/3%K, and (h) reduced 0.5%Ru/Al_2_O_3_/3%K.

The TEM micro images of the unpromoted and promoted 0.5%Ru/Al_2_O_3_ catalyst nanostructures are summarized and discussed below. Some RuO_2_ and metallic Ru particles formed on fresh and reduced catalyst samples respectively were illustrated by the red circles on TEM images. Fig. 5 shows the TEM data for the alumina-supported catalysts.

Fig. 5a suggests that the unpromoted 0.5%Ru/Al_2_O_3_ catalyst is a fine crystalline nanostructured material with an average particle size of 2.5 nm for RuO_2_. When the 0.5%Ru/Al_2_O_3_ catalyst sample was reduced at 230°C (Fig. 5b), the nanostructure of the catalyst was similar to the unreduced catalyst and Ru is seen in various areas of catalyst support. The addition of 1 % potassium transformed the nanostructure to become a bit agglomerated in some few areas of the catalyst support (Fig. 5c). The same effect was also observed on a sample reduced with hydrogen at 230 °C (Fig. 5d). A further increase in potassium loading to 2 % did not have a significant effect in the catalyst alteration (Fig. 5e). Similarly, the reduced sample also did not show any significant catalyst alteration with the addition of 2%K (Fig. 5f). When adding 3%K (Fig. 5g) the observations were similar to those discussed in Fig. 5e for 2%K addition and similarly on a reduced 3%K sample (Fig. 5h), no further catalyst alterations observed.

The above data explains FT results obtained whereby the unpromoted 0.5%Ru/Al_2_O_3_ catalyst (fine and crystalline) yielded better CO_2_ conversion though with a CH_4_ selectivity of 100 % (Fig. 8). However, when 1%K was added to the catalyst, the nanostructure became slightly agglomerated, which in turn allowed for a slight formation of C_2+_ hydrocarbons albeit at slightly reduced CO_2_ conversion. Further addition of potassium agglomerated Ru particles more and hence FT performance did not improve (Fig. 16).

#### 3.1.5 X-ray photoelectron spectroscopy (XPS) analysis

**Fig. 6:**
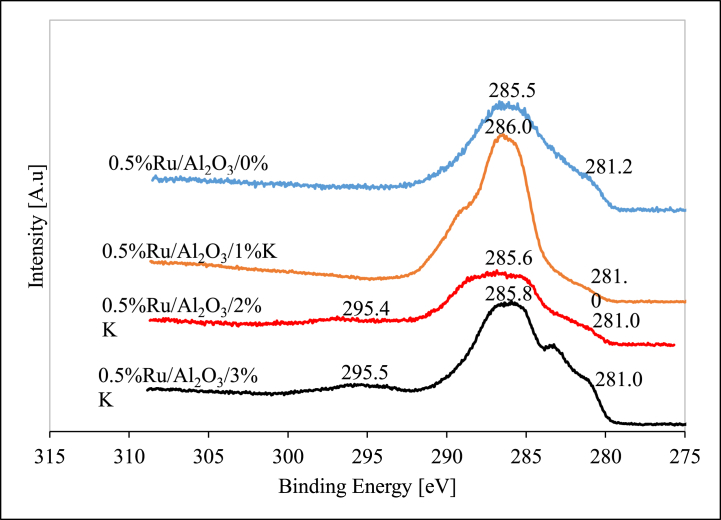
XPS data for fresh (before reduction) alumina-supported Ru catalysts.

**Fig. 7:**
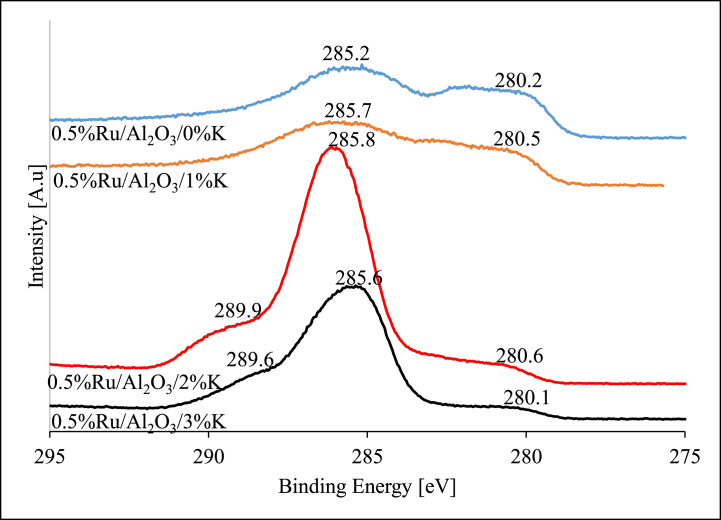
XPS data for reduced alumina-supported Ru catalysts.

The XPS profiles for the unpromoted as well as the K-promoted alumina-supported Ru catalysts are summarized in Fig. 6 (before reduction) and Fig. 7 (after reduction).

**Fig. 8:**
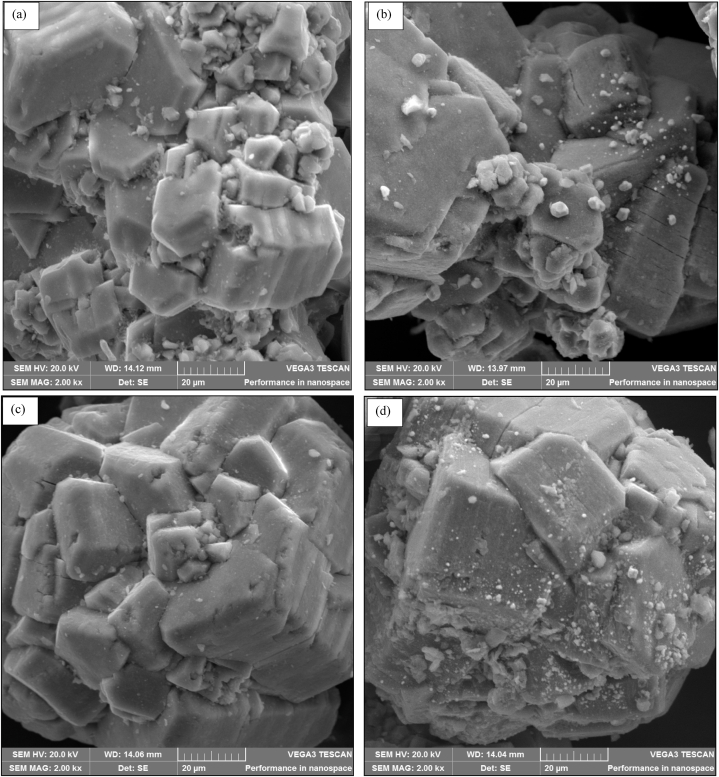
SEM data for (a) fresh 0.5%Ru/Al_2_O_3_, (b)fresh 0.5%Ru/Al_2_O_3_/1%K, (c) fresh 0.5%Ru/Al_2_O_3_/2%K, and (d) fresh 0.5%Ru/Al_2_O_3_/3%K.

The Ru 3d was observed in all fresh alumina supported catalysts as seen in Fig. 6. The Ru 3d_5/2_ peak was observed at a binding energy of 281.2 eV for the unpromoted catalyst while there was no potassium peak detected as expected [11]. The binding energy of RuO_2_ remained around 281 eV across all different variants of catalyst. When 1%K was added to the catalyst and K 2p_3/2_ peak was not clearly visible, however, it was detected at 295.4 eV for 2%K catalyst and 295.6 eV for 3%K catalyst sample. This could indicate that 1%K is so minute and completely dissolved in support and forming strong interactions with it while at further K additions, catalysts samples become more agglomerated, and K is also available in excess. Theoretical BE value for K 2p is 294.6 eV which is in line with the figured obtained in this work, (Moulder et al. (1992:225)). The Ru 3d_3/2_ was also detected at BE of 285.5 eV for 0%K, 286.0 eV for 1%K, 285.6 eV for 2%K and 285.8 eV for 3%K. The Ru 3d_5/2_ represents RuO_2_ while the Ru 3d_3/2_ is attributed to RuO_3_. The XPS profiles for the unpromoted as well as the K-promoted alumina supported Ru catalysts (after reduction) are summarized in Fig. 7.

All reduced alumina supported ruthenium-based catalysts followed a similar trend to that of the fresh calcined catalyst samples (Fig. 7). The Ru 3d_5/2_ were observed at BE of 280.2, 280.5, 280.6, and 280.1 eV for the unpromoted, 1%K, 2%K, 3%K respectively. This peak is attributed to Ru^0^ since theoretical BE value is around 280.1 eV. The BE on this peak seemed very consistent and repeatable in all samples. A large peak at BE ranging from 285.2 to 285.8 eV was present in all samples denoting Ru 3d_5/2_. Once again, the K peak was not very evident for 1%K catalyst sample, and it was detected at 289.9 and 289.6 eV for 2%K and 3%K catalyst samples respectively.

#### 3.1.6 Scanning electron microscopy (SEM)

The SEM micro images of unpromoted and promoted 0.5%Ru/Al_2_O_3_ catalysts are shown in Fig. 8. The data shows the morphology of the 0.5%Ru/Al_2_O_3_ (Fig. 8a), and potassium promoted samples (Fig. 8b–(d)). The unpromoted sample demonstrated a smooth and compact morphology with some few small porous particles scattered around. The addition of potassium significantly filled the pores in the small particles surface and the gaps between adjacent Ru resulting in improved interconnectivity of the particles composing Ru (Fig. 8b). Further addition of potassium, however, transformed the structure of this catalyst to become highly compacted solid (Fig. 8c and (d)). This could explain the results of catalysts performance evaluation as higher CO_2_ conversion was achieved with unpromoted catalyst and this conversion started declining as potassium was added and clogged some pores and making the catalyst too compact to enhance chemical reaction. The improved interconnectivity of particles at 1%K seems to almost alter product selectivity slightly as seen with small yield of C_2+_ hydrocarbons from catalyst activity tests done. As seen with catalyst morphologies on Fig. 8c and (d), catalysts have become more compact and hence the poor performance in activity tests in terms of CO_2_ conversion and hydrocarbon product yield. This suggests that a further addition of K beyond 1 % makes performance of these catalysts worse, as it fills the pores and hence reduces the uptake of reactants.

#### 3.1.7 Energy dispersive X-ray spectroscopy (EDX)

Energy dispersive x-ray spectroscopy was conducted to identify elements contained in prepared catalysts and to determine homogeneity and elemental distribution in the synthesized structure. This was also done to understand the physical interaction of K with Ru and/or the support.

During EDX measurement, different samples were focused, and the corresponding peaks are shown in Fig. 10a–d. Ruthenium can be seen in unpromoted sample (Fig. 10a), and both Ru and K can be seen in promoted samples (Fig. 10b–d) of the synthesized composite nanostructure in the EDX spectrum. The quantities of Ru, Al, K and O measured in weight percentages and summarized in Table 2.

**Table 2:** EDX weight ratio of electrospun (0.5%Ru/Al_2_O_3_) nanocomposite.

Composite	Ruthenium (Ru) Weight %	Aluminium (Al) Weight %	Potassium (K) Weight %	Oxygen (O) Weight %
0.5%Ru/Al_2_O_3_/0%K	2.4	39.9	0	38.9
0.5%Ru/Al_2_O_3_/1%K	9.6	30.7	0.2	40.3
0.5%Ru/Al_2_O_3_/2%K	1.9	35.4	0.6	41.9
0.5%Ru/Al_2_O_3_/3%K	8.7	36.7	0.8	30.8

**Fig. 9:**
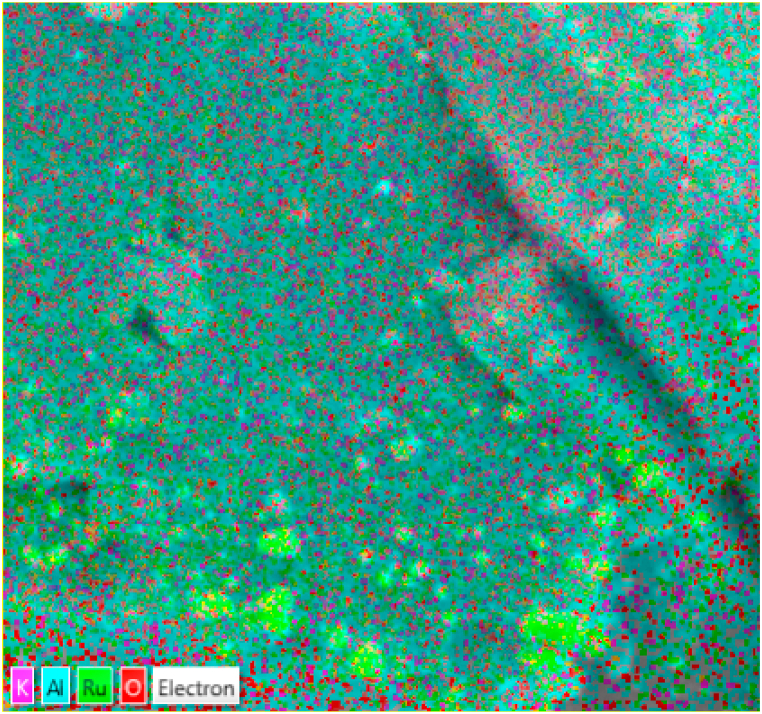
EDS layered image of 0.5Ru/Al_2_O_3_/1%K.

Carbon was also present in all composite materials, but it was not considered on the results as all materials were carbon-coated to improve their conductivity only to enable the analysis. The data in Table 2 confirm that Ru was present in all prepared catalysts and K was also present in promoted catalyst samples. No foreign materials could be detected except carbon which was used to carbon-coat the samples for SEM and EDX analysis. The layered image of K promoted sample of this catalyst are presented in Fig. 9.

**Fig. 10:**
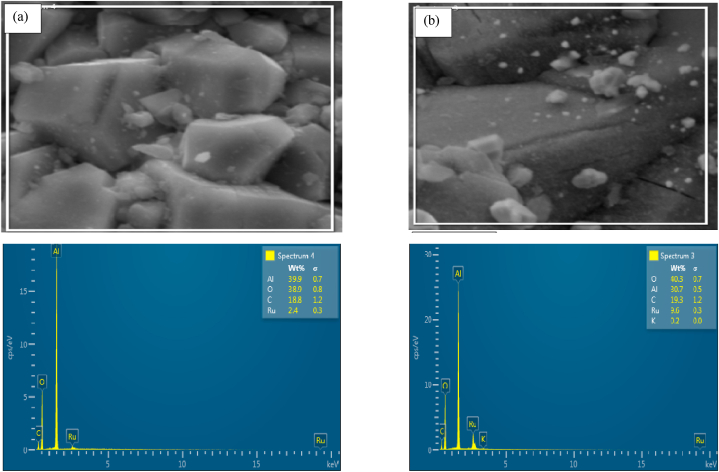

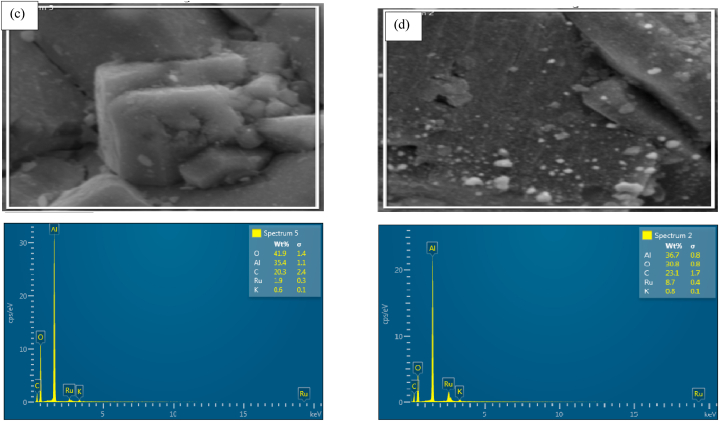
EDX data for (a) fresh 0.5%Ru/Al_2_O_3_, (b)fresh 0.5%Ru/Al_2_O_3_/1%K, (c) fresh 0.5%Ru/Al_2_O_3_/2%K, and (d) fresh 0.5%Ru/Al_2_O_3_/3%K.

The EDS layered image (Fig. 9) demonstrates that both Ru and K are evenly distributed on alumina support. There are, however, some marks showing agglomerated alumina on the composite surface. There is some good interaction between K and Ru. This is clearly visible on some agglomerated Ru elements of the composite structure.

### 3.2 Catalyst evaluation

#### 3.2.1 Effect of temperature

**Fig. 11:**
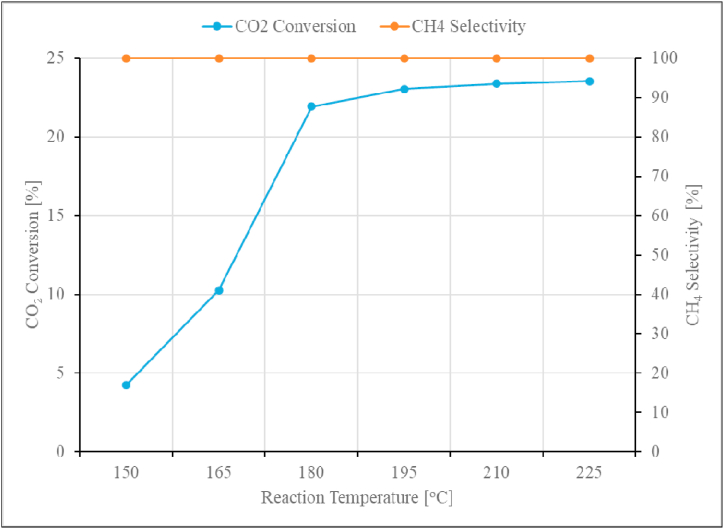
CO_2_ conversion and CH_4_ selectivity as a function of reaction temperature over unpromoted Ru/Al_2_O_3_ catalyst. Reaction conditions: Pressure: 1 bar, Space velocity: 1.2 Nl/gCat/h.

The effect of reaction temperature was studied using the unpromoted Ru/Al_2_O_3_ catalyst in the range of 150–225 °C and the data are shown in Fig. 11.

The CO_2_ conversion over the unpromoted Ru/Al_2_O_3_ catalyst was observed to increase with increasing temperature and seems to reach the highest of 23 % at 195°C, beyond this point, rate of CO_2_ conversion slowed down. The product formed was exclusively CH_4_ over the entire range of temperature used in this study with no formation of other hydrocarbons. This behaviour with increasing CO_2_ conversion trend with increasing temperature is better explained by Sebatier reaction theory of CO_2_ methanation. A similar trend for CO_2_ conversion, though in a higher temperature range (250–410 °C) was also observed in the earlier study [12]. A similar trend on CO_2_ conversion over alumina-supported ruthenium catalyst was also observed in literature, however, with different product selectivity [4]. The study also reported the formation of small amounts of C_2_H_2_ that slightly increased with an increasing temperature and CH_3_OH which decreased with increasing temperature [4]. The observations on selectivity of CH_4_ from this study, were more in line with the finding reported in the literature which used temperature range of 175–325 °C, an H_2_:CO_2_ ratio of 4:1, and a Ru loading of 0.5 % on Al_2_O_3_ [13]. The temperature of 195 °C was retained as optimum for the rest of the study. There is potential to form other hydrocarbons if the operating conditions (feed composition and reactor catalyst loading) used in this study could be altered [4].

The activation energy (Ea) of CO_2_ methanation reaction was obtained using the Arrhenius equation described as equation (2) below.

(2) $$k={Ae}^{\frac{E_{a}}{RT}}\;\text{or}\;\ln\;k=-\frac{E_{a}}{RT}+\ln\;A$$

where:

k = chemical reaction rate.

A = pre-exponentian factor.

E_a_ = Activation energy (J/mol).

R = Gas constant.

T = Temperature (K).

**Fig. 12:**
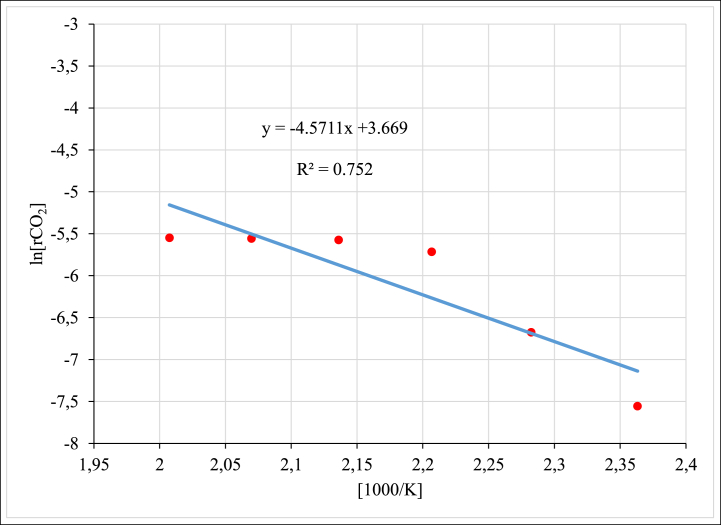
Arrhenius Plot of CO_2_ methanation reaction over unpromoted Ru/Al_2_O_3_ catalyst.

The Arrhenius plot (Fig. 12) shows a near linear trend which is attributed to the dominance of a kinetic regime [8]. Therefore, by applying the Arrhenius equation on the data, the activation energy of CO_2_ hydrogenation reaction was calculated using the slope of the Arrhenius plot in Fig. 12 to be 38 kJ/mol. This activation energy is significantly lower than what is reported in the literature [8], where it was reported to be 73.3 kJ/mol for CO_2_ hydrogenation over Ru/Al_2_O_3_ catalyst. The difference could be due to the amount of ruthenium loaded in the alumina of 1 % compared to 0.5 % used in the current study and the CO_2_:H_2_ feed ratio of 1:4 compared to 1:3 used in this study respectively. On the other hand, the activation energy (E_a_) in the range of 57–70 kJ/mol using the temperature range of 240–300 °C for 0.5%Ru/gAl_2_O_3_ catalyst was also reported [12] and is more in line with the findings in this study on the lower operating temperature range of 150–225 °C.

#### 3.2.2 Effect of pressure

**Fig. 13:**
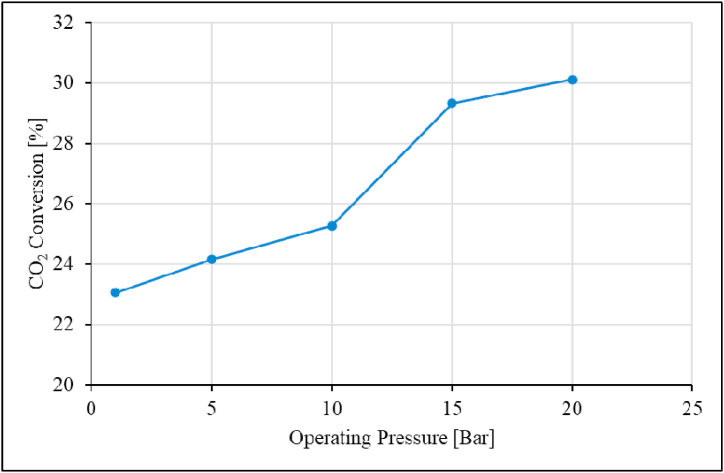
CO_2_ conversion as a function of operating pressure over unpromoted Ru/Al_2_O_3_ catalyst. Reaction conditions: Temperature: 195 °C, Space velocity: 1.2 Nl/gCat/h.

**Fig. 14:**
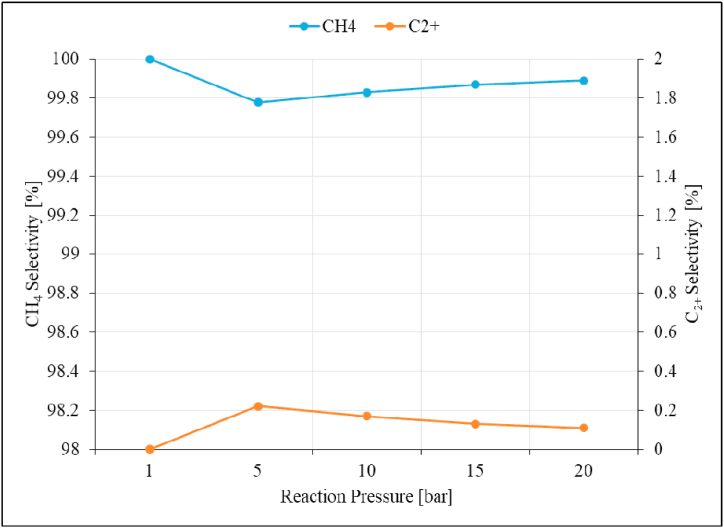
CH_4_ and C_2+_ Selectivity as a function of reaction pressure over unpromoted Ru/Al_2_O_3_ catalyst. Reaction conditions: Temperature: 195 °C, Space velocity: 1.2 Nl/gCat/h.

**Fig. 15:**
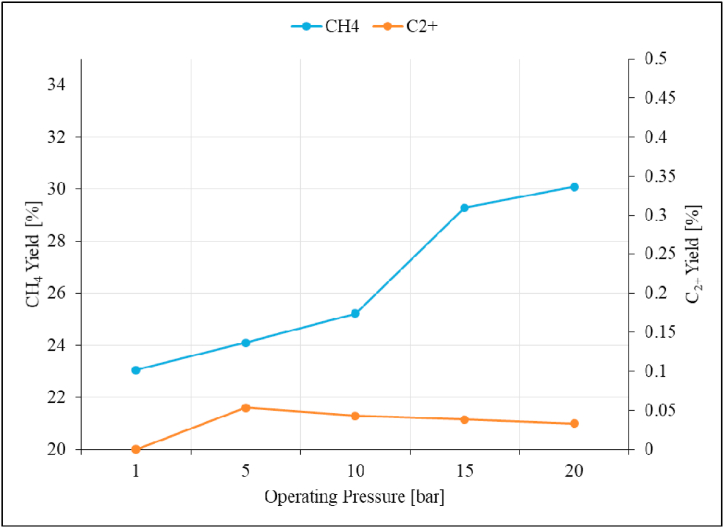
CH_4_ and C_2+_ product yield as a function of reaction pressure over unpromoted Ru/Al_2_O_3_ catalyst. Reaction conditions: Temperature: 195 °C, Space velocity: 1.2 Nl/gCat/h.

The effect of operating pressure was studied in the pressure range of 1–20 bar; the results for the unpromoted Ru/Al_2_O_3_ catalyst are presented in Fig. 13, Fig. 14, Fig. 15.

**Fig. 16:**
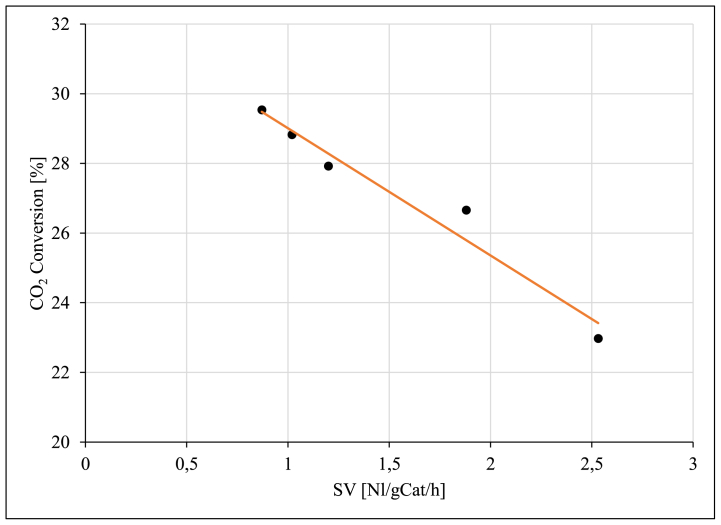
CO_2_ conversion as a function of space velocity (SV) over unpromoted Ru/Al_2_O_3_ catalyst. Reaction conditions: Temperature: 195 °C, Pressure: 5 bar.

The data shows that the CO_2_ conversion increases gradually from 23 to 25 % with increasing pressure from 1 bar to 10 bar respectively. From 10 to 15 bar, CO_2_ conversion rapidly increases from 25 to 29 %, and then slows down to 30 % at 20 bar. This was attributed to the overall molecular increase as the operating pressure is increased. The increased CO_2_ conversion continues until equilibrium is reached at which point the CO_2_ conversion plateaus. This is in line with what has been reported in previous studies on zirconia-supported ruthenium catalysts on CO_2_ hydrogenation [14,15]. Corresponding data for CH_4_ and C_2+_ selectivity was presented in Fig. 14.

C_2+_ hydrocarbons were observed at increased reaction pressures of 5 bar and then decreases gradually with further pressure increase. In this pressure range, CH_4_ decreased to the lowest value of 99.78 % at 5 bar, while small amounts of C_2+_ hydrocarbons (0.22 %) were formed at the same pressure. Increasing reaction pressures beyond 5 bar did not improve C_2+_ hydrocarbons selectivity and no C_2+_ hydrocarbons were detected on similar studies [12,14]. CH_4_ and C_2+_ product yields are presented in Fig. 15.

C_2+_ product yield follows a similar trend to that of the selectivity observed previously on Fig. 14. There is a notable rise in product yield of C_2+_ hydrocarbons to 0.05 % at 5 bar, followed by steady decline beyond this point reaching 0.03 % at 20 bar. On the other hand, CH_4_ product yield rises slowly at first from 0 to 10 bar where it rapidly increases and beyond this point reaching maximum of 30 % at 20 bar. Based on these observations, the operating pressure of 5 bar was selected as optimal for the rest of the study. This pressure was selected on the basis that it showed slight-increased product yield for C_2+_ which is one of the hydrocarbon products this study is aiming to achieve instead of methane.

#### 3.2.3 Effect of space velocity (SV)

**Fig. 17:**
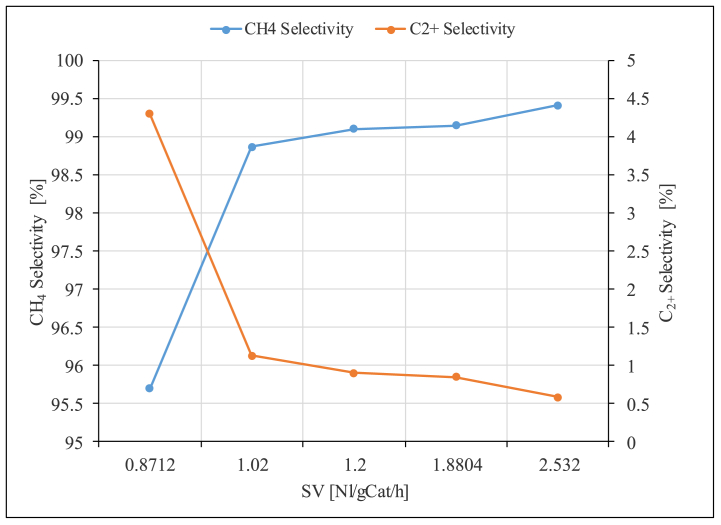
CH_4_ and C_2+_ selectivity as a function of space velocity (SV) over unpromoted Ru/Al_2_O_3_ catalyst. Reaction conditions: Temperature: 195 °C, Pressure: 5 bar.

**Fig. 18:**
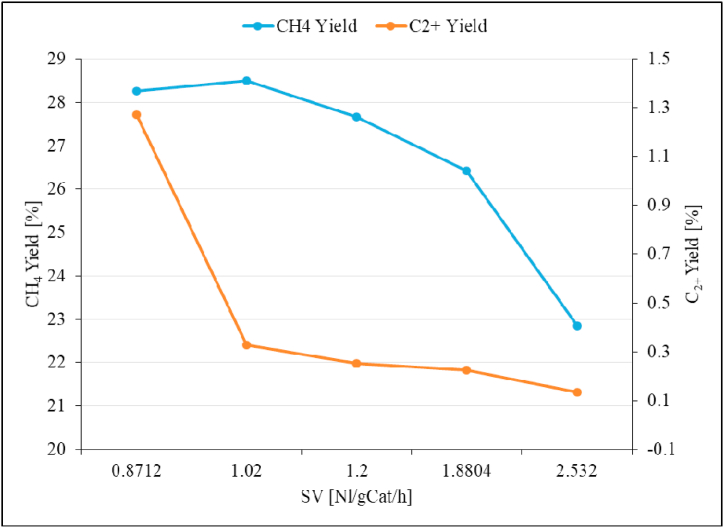
CH_4_ and C_2+_ yield as a function of space velocity (SV) over unpromoted Ru/Al_2_O_3_ catalyst. Reaction conditions: Temperature: 195 °C, Pressure: 5 bar.

The effect of space velocity (SV) on the reaction product formation and the CO_2_ conversion was studied using the unpromoted Ru/Al_2_O_3_ catalyst. The results obtained are presented in Fig. 16, Fig. 17, Fig. 18.

The CO_2_ conversion decreased almost linearly with increasing space velocity from 29.5 % (at 0.87 Nl/gCat/h) to 22.9 % (at 2.53 Nl/gCat/h) at 195 °C [12]. The decreasing CO_2_ conversion as the space velocity was increasing was expected since reduction in residence time of reactants is reduced at high space velocities.

Fig. 17 shows that the CH_4_ selectivity sharply increases from 95.7 % to 98.9 % as the space velocity was raised from 0.87 Nl/gCat/h to 1.02 Nl/gCat/h and then no significant change was observed as the SV was increased further. The corresponding C_2+_ selectivity on the other hand decreased significantly from 4.3 % to 1.1 % with no significant change as the SV is increased further to 2.53 Nl/gCat/h. A different product distribution with declining CH_4_ selectivity with increasing SV but with less pronounced effects was reported in literature [12]. There was no formation of other hydrocarbons in reported literature, but CO selectivity increased up to 0.5 % at the highest SV studied [12]. Once again, the difference in product selectivity could be a result of elevated reaction temperature (310 °C compared to 195 °C used in this study) which indicates that higher reaction temperatures favors CH_4_ formation over long chain hydrocarbons. Interesting observation also is that CH_4_ yield follows a similar trend to CO_2_ conversion (Fig. 18).

The CH_4_ product yield gradually declines with increasing SV, while C_2+_ product yield drops sharply from 1.27 % to 0.33 % when the SV was increased from 0.87 Nl/gCat/h to 1.02 Nl/gCat/h and no significant changes beyond this point as the SV increase further. Based on the above observations, an optimum space velocity of 0.87 Nl/gCat/h was selected for the rest of the experiments in this study.

#### 3.2.4 Effect of potassium loading

**Fig. 19:**
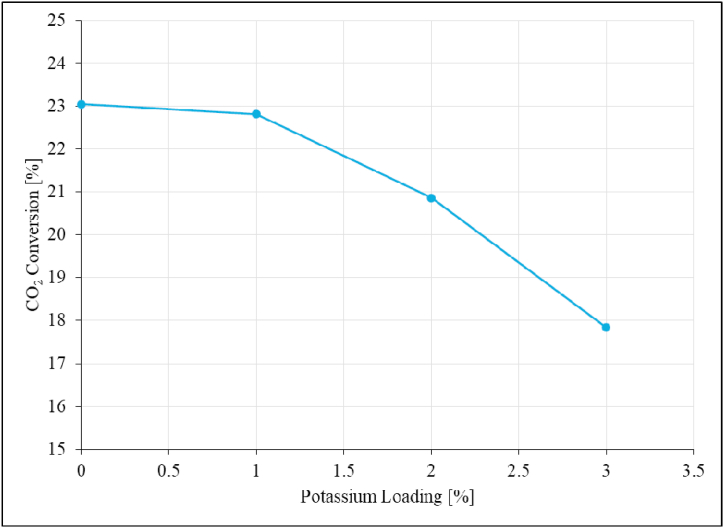
CO_2_ conversion as a function of potassium loading over Ru/Al_2_O_3_ catalyst. Reaction conditions: Temperature: 195 °C, Pressure: atmospheric, and SV: 0,87 Nl/gCat/h.

**Fig. 20:**
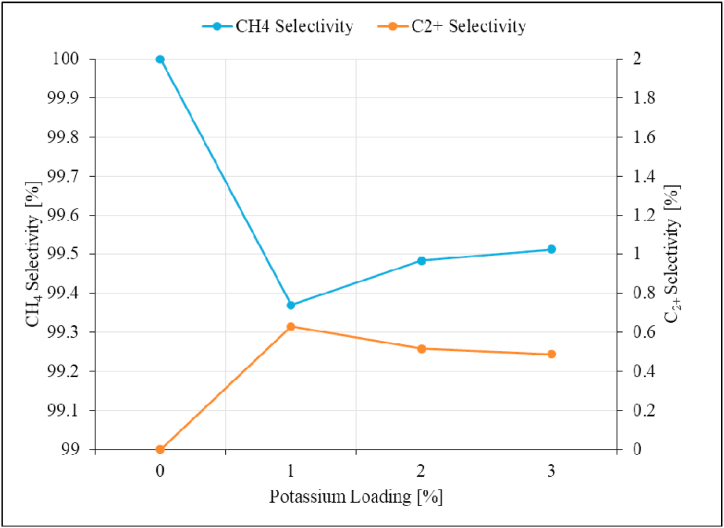
Product selectivity as a function of potassium loading over Ru/Al_2_O_3_ catalyst. Reaction conditions: Temperature: 195 °C, Pressure: atmospheric, and SV: 0,87 Nl/gCat/h.

**Fig. 21:**
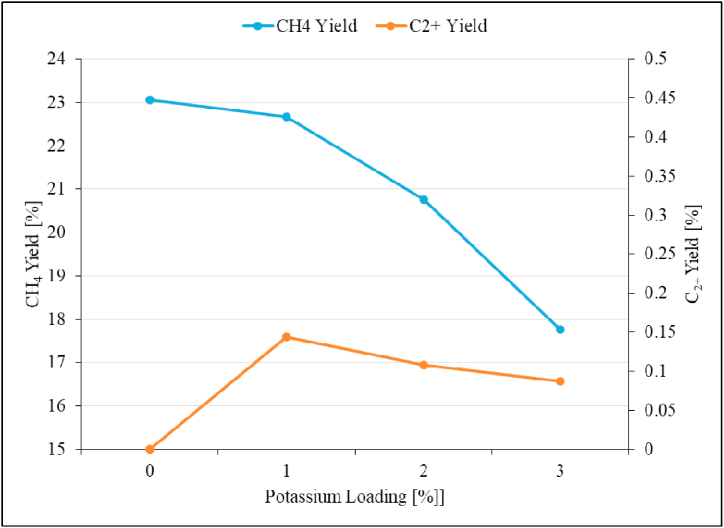
Product yield as a function of potassium loading over Ru/Al_2_O_3_ catalyst. Reaction conditions: Temperature: 195 °C, Pressure: atmospheric, and SV: 0,87 Nl/gCat/h.

The effect of promoter (potassium) was studied at 195 °C and atmospheric pressure with Ru/Al_2_O_3_ catalyst promoted with 1–3 % potassium. The results are presented in Fig. 19, Fig. 20, Fig. 21.

The addition of potassium as a promoter reduced CO_2_ conversion slightly from 23 % to 22.8 % when the catalyst was promoted with 1%K (Fig. 19). Increasing potassium loading further did not have significant effect beyond 1%K with regard to CO_2_ conversion. These findings are in agreement with what has been reported in the earlier study [5]. This could be explained by catalysts becoming more agglomerated when K is added and filling some pores and spaces between Ru particles as seen on SEM images. Fig. 20 shows that the addition of K to Ru-based catalysts promotes the formation of C_2+_ hydrocarbons.

While the product of CO_2_ hydrogenation over the unpromoted Ru/Al_2_O_3_ catalyst was exclusively methane, promoting the catalyst with 1%K led to a C_2+_ hydrocarbons selectivity of 0.63 % and then stabilized around this value as potassium loading was increased up to 3 %. The C_2+_ product yield increases from 0 to 0.14 % when 1%K was added (Fig. 21), and then no improvement with regards to C_2+_ yield as potassium content was increased further up to 3 %.

The CH_4_ yield however, significantly decreased from 23 % to 17.76 % with increasing potassium content from 0 to 3 % [5]. It is evident from the above trends and discussions that the optimum potassium loading for improved C_2+_ product yield over Ru/Al_2_O_3_ is 1%K for CO_2_ hydrogenation. At 1%K there was increase in C_2+_ yield while the CH_4_ product yield significantly decreased.

## 4 Conclusions

The main purpose of this study was to investigate the catalytic hydrogenation of CO_2_ into hydrocarbons over ruthenium-based catalysts. Data observed showed that the product formed from CO_2_ hydrogenation was predominantly methane with Ru proving to be extremely active even without any promoters. When potassium was added as a promoter to the catalyst, some C_2+_ hydrocarbons started to form. However, increasing the K loading to higher levels had no further beneficial effects. The optimum potassium loading for improved C_2+_ product yield over Ru/Al_2_O_3_ was 1%K for CO_2_ hydrogenation. TEM data revealed that addition of potassium changes the structure of catalyst since the Ru particles agglomerated together in several locations on the surface of the support material (alumina). This agglomeration increased as K loading was increased, leading to CO_2_ conversion deteriorating. This was further supported by TEM images which revealed that Ru is somewhat evenly distributed on the support for the unpromoted catalysts sample as compared to clustering observed on K promoted samples. The SEM micro images reveal that alumina-supported catalyst appear smooth on surface, but compact in structure, and less porous. Although the activity of 0.5%Ru/Al_2_O_3_/1%K showed the greater benefit from K addition, the hydrocarbon yield was still dominated by CH_4_ with no significant hydrocarbons chain growth, and this suggests that Ru-based catalysts supports methanation process. It is recommended to explore other promoters on 0.5%Ru/Al_2_O_3_ to improve long chain hydrocarbons production yield. The effect of Ru loading to be investigated at optimal process conditions determined in this study. This will provide some understanding of whether larger Ru particles could enhance chain growth or not. The effect of the feed composition needs to be explored as well. This will give an insight into catalyst performance at varied feed composition.

## CRediT authorship contribution statement

**Galeboe Ralengole:** Writing – original draft, Visualization, Methodology, Investigation, Funding acquisition, Formal analysis, Data curation, Conceptualization. **Kalala Jalama:** Writing – review & editing, Supervision, Project administration, Conceptualization. **Phathutshedzo Khangale:** Writing – review & editing, Supervision, Resources, Project administration, Methodology, Conceptualization.

## Declaration of competing interest

The authors declare that they have no known competing financial interests or personal relationships that could have appeared to influence the work reported in this paper.
